# Potential use of Chinese chive (Allium tuberosum) extract as a natural antimicrobial agent against Escherichia coli in lightly pickled vegetables

**DOI:** 10.1099/acmi.0.001073.v3

**Published:** 2026-04-10

**Authors:** Satoko Miya

**Affiliations:** 1 Faculty of Nutrition, Tokyo Kasei University, 1-18-1, Kaga, Itabashi, Tokyo 173-8602, Japan

**Keywords:** Chinese chive, *Escherichia coli*, lightly pickled vegetable, napa cabbage

## Abstract

Lightly pickled vegetables have gained popularity as a healthier alternative to traditional high-salt pickles; however, their low salt content increases the risk of foodborne pathogen proliferation. In this study, we aimed to investigate the antibacterial effects of Chinese chive extract (CCE) against *Escherichia coli* using culture media and lightly pickled napa cabbage as model systems. The results revealed that CCE exhibited concentration-dependent inhibitory effects on *E. coli* growth. On agar media, ≥3% CCE completely suppressed colony formation. In broth media, ≥3% CCE clearly inhibited *E. coli* proliferation at both 10 and 35 °C. Furthermore, in lightly pickled cabbage treated with CCE, the growth of coliform and total aerobic bacteria was significantly suppressed at both refrigeration and ambient temperatures. Thus, CCE could serve as a promising natural antimicrobial agent in the production of lightly pickled vegetables, offering a strategy to potentially enhance food safety, independent of synthetic food additives.

## Data Summary

All data associated with this work are reported within the article and the supplementary file.

## Introduction

Fresh vegetables are important for human health, since they provide nutrients such as vitamins, minerals and fibre in their unaltered forms. Pickled vegetables offer an easy and convenient approach to promote vegetable intake. However, traditional pickles are not preferred due to their high salt content. Recently, lightly pickled vegetables containing ∼2% sodium chloride (NaCl) have gained popularity as a relatively healthier alternative. Nevertheless, the reduced salt content may allow the growth of bacteria, including serious foodborne pathogens.

Lightly pickled vegetables have caused several foodborne outbreaks in Japan. For example, in 2012, lightly pickled napa cabbage caused a large-scale *Escherichia coli* O157:H7 outbreak, affecting 169 individuals, including eight fatalities [1,2]. Several other food poisoning incidents caused by lightly pickled vegetables have been reported, including those due to turnip in 2000 [3], cucumber in 2002 and 2016 [4,5] and eggplant and perilla in 2011 [5]. All of these outbreaks in Japan have been caused by enterohaemorrhagic *E. coli*.

Raw vegetables are frequently contaminated with foodborne pathogens. Since such contaminants are often difficult to eliminate via washing and sanitizing, it is imperative to control bacterial growth throughout the processing and preservation of lightly pickled vegetables. Although various chemicals have been used to reduce bacterial growth in vegetables [6,7], the use of synthetic food additives is often discouraged owing to growing health consciousness. Consequently, the use of natural substances to suppress microbial growth is considered a more favourable approach.

Various natural plant extracts exhibit antimicrobial activities [8]. Chinese chives (*Allium tuberosum*), popularly consumed raw or cooked particularly in Asian countries, contain various phytochemicals exhibiting antimicrobial properties [9,12]. Moreover, these compounds also potentially confer health benefits to humans [13,14].

Accordingly, this study aimed to evaluate the antibacterial potential of Chinese chive extracts (CCEs) in lightly pickled vegetables. Since enterohaemorrhagic *E. coli* O157:H7 is the most prevalent bacterium causing food poisoning in lightly pickled vegetables, we conducted this study using *E. coli* as a model organism.

## Methods

### Bacterial strains

Three *E. coli* strains, IAM1264 (Institute of Applied Microbiology, University of Tokyo), NBRC3972 (NITE Biological Resource Center) and NBRC102203, were used in this study. The strains were grown overnight in Tryptic soy broth (TSB; Becton Dickinson, Franklin Lakes, NJ, USA) at 35 °C before being used for the experiments.

### Preparation of sterile CCE

Chinese chives were obtained from local grocery stores and used within 24 h of purchase. Ten centimetres of the lower stem portion of the Chinese chives was trimmed for use, as this portion is generally considered inedible, and its use will help reduce food waste. Moreover, our preliminary experiments have suggested that the lower stem of Chinese chives possesses antimicrobial activity comparable to that of other plant parts (data not shown). The Chinese chives were finely chopped, and an equal w/v amount of PBS (for culture medium experiments) or distilled water (DW; for pickled napa cabbage experiments) was added; the mixture was ground using a kitchen blender. The ground Chinese chives were then centrifuged at 15,000***g*** for 10 min, and the supernatant was sequentially sterilized using filters with pore sizes of 0.45 and 0.22 µm (Merck, Darmstadt, Germany). This sterile CCE was used in the following experiments.

### Experiments on agar plates

Tryptic soy agar (TSA; Becton Dickinson) was prepared with varying CCE concentrations. When preparing the TSA plates, the volume of DW was reduced by a predetermined amount. Following autoclave sterilization, an equivalent volume of CCE was added prior to solidification to obtain TSA with 0%, 1%, 3%, 5%, 8% and 10% CCE concentrations. After solidification, 50 µl of each of the three *E. coli* strain cultures (∼10^4^ c.f.u. ml^−1^) was separately streaked onto TSA plates using a spiral plater (Eddy Jet 2, IUL, S.A., Barcelona, Spain), followed by incubation for 48 h at 35 °C for cell enumeration.

### Experiments in broth culture

The TSB was prepared at the same CCE concentrations as those for agar plates (0–10%). The reduced amount of DW was replaced with CCE following autoclave sterilization. Furthermore, 100 µl of each of the three *E. coli* strain cultures (∼10^6^ c.f.u. ml^−1^) was added to TSB and incubated at 10 °C for 7 days or at 35 °C for 6 h. In the experiment at 35 °C, 50 µl of the samples was inoculated onto TSA plates every hour, using a spiral plater, to determine the bacterial counts, whereas in the experiment at 10 °C, inoculation onto TSA plates was conducted on days 0, 1, 3, 5 and 7.

### Experiments with lightly pickled napa cabbage

Napa cabbage is a commonly used vegetable for making pickles; hence, it was evaluated in this study. Napa cabbage was purchased from a local grocery store, cut into ∼2 cm square sections, immersed in a 100 p.p.m. sodium hypochlorite solution for 10 min and rinsed with sterile DW. Furthermore, 2% NaCl was added, and the cabbage was gently massaged for 30 s. The napa cabbage was divided into 10 g portions, and each portion was inoculated with 1 ml of a mixed suspension of the three *E. coli* strains, which had been serially diluted to ∼10^4^ c.f.u. ml^−1^. After inoculation, 1, 5 or 10 ml of CCE was added to each portion, while samples without CCE were used as negative controls. After incubation at 10 °C for 7 days or at 25 °C for 12 h, 90 ml of PBS was added to each sample, and the samples were homogenized using a Stomacher 400 homogenizer (Seward, London, UK). Total bacterial counts were determined using TSA plates, and coliform counts were determined using desoxycholate agar plates (Nissui Pharmaceutical, Tokyo, Japan). The experiments were conducted in triplicate on three different occasions.

### Statistical analyses

The growth behaviour of *E. coli* at different CCE concentrations was statistically compared using Student’s t-test [15], and a *P*-value of less than 0.05 was considered statistically significant.

## Results

### Experiments on agar plates

When the number of colonies on TSA without CCE (negative control) was normalized to the control (0% CCE), TSA containing 1% CCE showed 82–95% of the colony count. In contrast, no colonies were observed on TSA containing 3–10% CCE (Table 1).

**Table 1.** Growth of *E. coli* on TSA plates containing various concentrations of CCE

Bacterial strain	Bacterial count (% of negative control)					
	CCE concn in TSA					
	Negative control (0%)	1%	3%	5%	8%	10%
IAM 1264	100	91	0	0	0	0
NBRC 3972	100	82	0	0	0	0
NBRC 102203	100	95	0	0	0	0

### Experiments in broth cultures

**Fig. 1.**
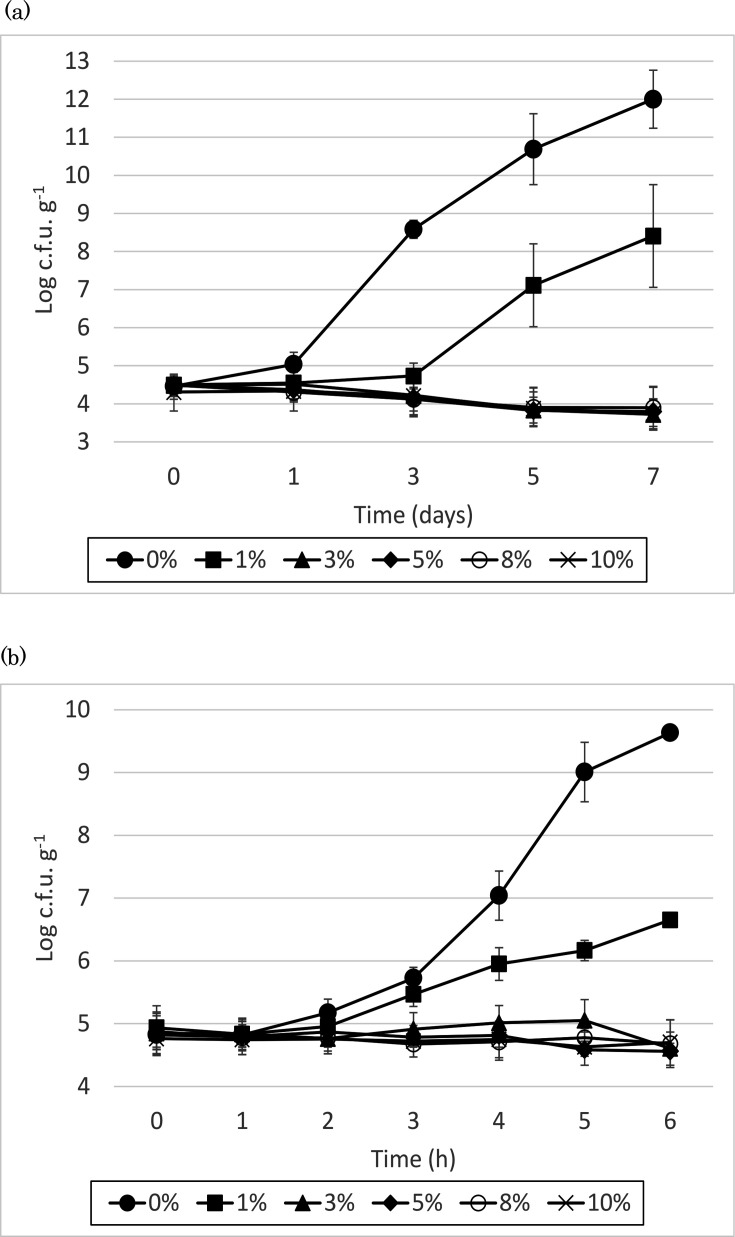
Growth behaviour of *E. coli* in TSB added with 0%, 1%, 3%, 5%, 8% or 10% sterile CCE. The *E. coli*-inoculated TSBs were incubated at 10 °C for up to 7 days (**a**), and at 35 °C for up to 6 h (**b**).

Results from the three bacterial strains were averaged, and the outcomes of incubation at 10 and 35 °C are shown in Fig. 1a, b, respectively. At both these incubation temperatures, the growth inhibitory effect of CCE was observed relative to the negative control. Although *E. coli* growth was observed with the addition of 1% CCE, the growth rate was slower than that of the control. Moreover, ≥3% CCE inhibited *E. coli* growth, with a modest reduction in bacterial counts. Significant differences from the control were detected at 10 °C on day 3 in all treatment groups. At 35 °C, significant differences were also observed at 3 (5–10%), 5 (3%) and 6 h (1–10%) (*P*<0.05).

### Experiments with lightly pickled napa cabbage

**Fig. 2.**
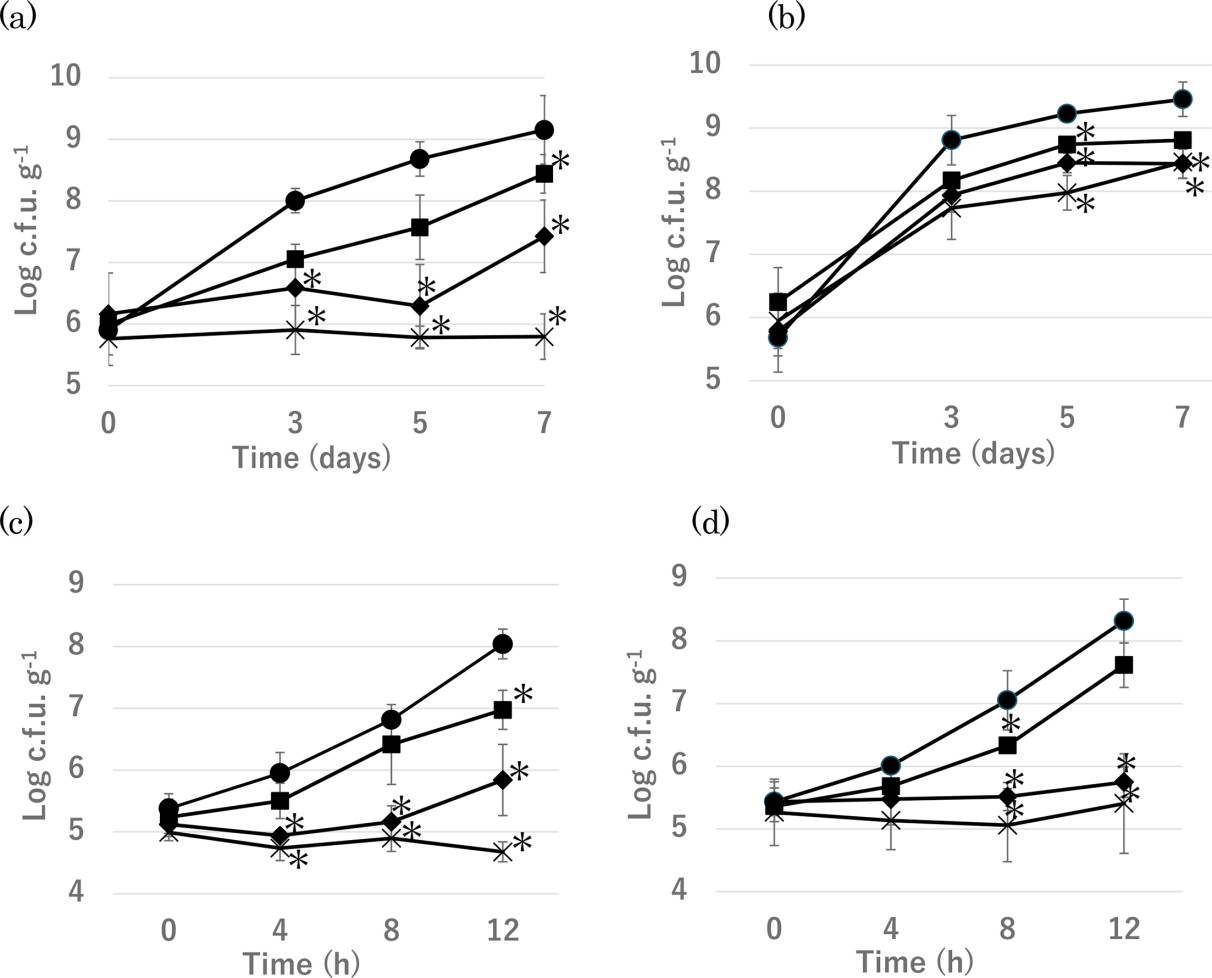
Growth behaviour of total coliforms (**a**, **c**) and aerobic bacteria (**b**, **d**) in lightly pickled napa cabbage at 10 and 25 °C, respectively. Samples were prepared with 0 (●), 1 (■), 5 (◆) or 10 ml (×) of CCE. An asterisk (*) indicates a statistically significant difference (*P*<0.05) relative to the negative control (CCE, 0 ml).

The growth of coliforms and total aerobic bacteria in napa cabbage treated with various CCE concentrations was evaluated in three independent experiments, and the average values are presented in Fig. 2. At both 10 °C for 7 days and 25 °C for 12 h, the growth of coliform bacteria was inhibited in a CCE concentration-dependent manner (Fig. 2a, c). In the samples treated with 1% CCE, a significant difference from the negative control was observed on day 7 at 10 °C and after 12 h at 25 °C. In contrast, in the samples treated with 5% and 10% CCE, significant differences from the negative control were observed from day 3 (10 °C incubation) and after 4 h (25 °C incubation), respectively. Furthermore, in the 10% CCE-treated samples, negligible bacterial growth was observed, and even a marginal decrease in the number of coliform bacteria was confirmed in the experiment conducted at 25 °C.

Regarding total aerobic bacteria, growth at 25 °C was inhibited in a concentration-dependent manner by CCE, as observed with coliform bacteria (Fig. 2d). Notably, in the samples treated with 5% and 10% CCE, little or no bacterial growth was observed during the 12 h incubation period. A similar concentration-dependent growth inhibition was observed at 10 °C; however, during the 7-day incubation period, total aerobic bacteria still increased by 3.1 log with 1% CCE, 2.9 log with 5% CCE and 2.8 log with 10% CCE (Fig. 2b).

## Discussion

Fresh vegetables harbour various types of bacteria, including potential pathogens [16]. In particular, *E. coli* is prevalent on vegetables and has caused disease outbreaks globally [17]. However, contamination may arise from various sources, including pre- and post-harvest, such as water [18,19], manure [20], wild animal faeces [21], insects, such as flies [22], and unsanitary handling [23]. Therefore, preventing contamination remains a significant challenge. Accordingly, the prevention of pathogenic bacterial growth on vegetables is essential.

Lightly pickled vegetables have become widely distributed recently due to their low salt content, making them healthier alternatives to traditional pickles. However, due to the frequent occurrence of foodborne illnesses caused by lightly pickled vegetables in Japan, the Ministry of Health, Labor and Welfare has introduced disinfection methods (such as sodium hypochlorite treatment or heat treatment) and low-temperature storage below 10 °C to the existing hygiene guidelines for pickled products in 2012. However, food poisoning caused by *E. coli* from lightly pickled vegetables still occurs; outbreaks linked to lightly pickled cucumbers resulted in 510 patients in 2014 [24] and 84 patients in 2016 [5].

Several studies have reported the bacteriostatic effects of Chinese chives on many bacterial strains, including *E. coli* [9,10, 13, 25]. Chen *et al*. [26] compared the antimicrobial activities of different parts of Chinese chives and found that the scape exhibits the most pronounced activity. However, since commercially available Chinese chives usually do not include the scape, the leaves, the second most active part, were used in this study, owing to their availability. In addition, since the bottom portion of the leaves is often discarded, we used this part for the experiments.

Experiments in this study using agar and broth media suggested that the CCE exhibited concentration-dependent bactericidal or bacteriostatic activity against *E. coli*. The experiments using broth media suggested that the addition of ≥3% CCE effectively inhibited the growth of *E. coli*, regardless of whether the incubation was conducted at 10 °C, as recommended in the hygiene guidelines for pickled products, or at 35 °C, which is favourable for *E. coli* proliferation. Thus, CCE use could serve as an effective and practical method for inhibiting bacterial growth in lightly pickled vegetables.

Furthermore, the experiment with lightly pickled napa cabbage was performed at the following two storage temperatures: 10 °C, as recommended by the guidelines of the Ministry of Health, Labor and Welfare in Japan described above, and room temperature (25 °C). In addition, the number of coliform bacteria, which are commonly used as hygiene indicator organisms in Japan, and the number of total aerobic bacteria were investigated. Consistently, the antimicrobial effect of the CCE was concentration dependent. When 10 ml of the extract was added to 10 g of napa cabbage, coliform growth was not observed during the experimental period, and the total aerobic bacteria count was significantly lower than that in the control group without the extract. While the suppression of coliform growth was successful, further reduction of total aerobic bacteria may require additional interventions. For instance, optimizing the pH of the extract, as suggested by Chen *et al*. [26], may further enhance its antimicrobial efficacy.

Although the relatively strong odour of Chinese chive may raise concerns at higher concentrations, Chinese chive is sometimes used as an ingredient in pickles; therefore, it may be acceptable depending on the product type and consumer preference.

## Conclusions

This study demonstrated the bacteriostatic and bactericidal effects of CCE against *E. coli*. The effect was concentration dependent in both artificial media and actual food matrices, such as lightly pickled napa cabbage. Notably, the addition of ≥3% CCE effectively inhibited *E. coli* growth under both refrigeration (10 °C) and ambient (25 °C) conditions, and also significantly suppressed total aerobic bacteria.

These findings highlight the potential of CCE as a natural antimicrobial agent in the production of lightly pickled vegetables, particularly in settings where strict temperature control is difficult to maintain. However, challenges such as the strong odour of Chinese chive and the limited reduction of total aerobic bacteria remain. Future studies should explore formulation improvements, such as pH adjustment or combination with other preservation methods, to enhance its practical applications in the food industry. Moreover, optimizing the sensory acceptability of CCE and enhancing its efficacy against a broader range of pathogens should be the focus of future research.

## Supplementary material

10.1099/acmi.0.001073.v3 Uncited Supplementary Material 1.

## Abbreviations

CCE: Chinese chive extract
DW: distilled water
TSA: Tryptic soy agar
TSB: Tryptic soy broth
